# Cadmium Stress Disrupts Auxin Signaling and Growth in *Ilex verticillata*: Insights from Physiological and Transcriptomic Analyses

**DOI:** 10.3390/plants15020277

**Published:** 2026-01-16

**Authors:** Qinyuan Shen, Liangye Huang, Piyu Ji, Muhammad Junaid Rao, Wanchun Li, Jianfang Zuo, Huwei Yuan, Daoliang Yan, Xiaofei Wang, Bingsong Zheng

**Affiliations:** 1 State Key Laboratory for Development and Utilization of Forest Food Resources, Zhejiang A&F University, Hangzhou 311300, China; 2024202011016@stu.zafu.edu.cn (Q.S.); 2023602121036@stu.zafu.edu.cn (L.H.); jipiyu123@163.com (P.J.); liwanchun@stu.zafu.edu.cn (W.L.); liangsie@zafu.edu.cn (D.Y.); xfwang@zafu.edu.cn (X.W.); bszheng@zafu.edu.cn (B.Z.); 2 Zhejiang Key Laboratory of Non-Wood Forest Products Quality Regulation and Processing Utilization, Zhejiang A&F University, Hangzhou 311300, China

**Keywords:** cadmium stress, *Ilex verticillata*, auxin signaling and IvMYB77-IvIAA interaction, oxidative stress, transcriptomics

## Abstract

Cadmium (Cd) pollution poses significant threats to ecosystems and human health, with agricultural soils in China particularly affected. *Ilex verticillata*, a popular ornamental plant, has not been extensively studied for its response to Cd stress. This study investigated the physiological and molecular mechanisms underlying Cd stress tolerance in *I. verticillata*, focusing on auxin signaling pathways. Under Cd stress (500 mmol/kg soil), *I. verticillata* exhibited inhibited stem growth, reduced photosynthetic capacity, and elevated oxidative stress markers such as malondialdehyde, H_2_O_2_, ·O_2_^−^, and antioxidant enzyme activities. Transcriptomic analysis revealed 3750 differentially expressed genes (DEGs) with significant enrichment in auxin signaling pathways. Six nucleus-localized *IvIAA* genes were identified and shown to interact with the transcription factor IvMYB77, suggesting a regulatory module in Cd stress responses. These findings highlight the role of auxin signaling in mediating Cd stress tolerance and provide insights into the molecular adaptation of *I. verticillata* to heavy metal pollution.

## 1. Introduction

The rapid advancement of industrialization has made environmental pollution a global challenge, among which heavy metal contamination is particularly critical. In China, cadmium (Cd) pollution severely affects agricultural soils, with an exceedance rate of 7% [1], establishing Cd as the country’s most problematic heavy metal pollutant. Characterized by high soil persistence and bioavailability, Cd poses significant threats to ecosystem integrity and human health. It disrupts normal growth and development in plants and can in the human body, potentially leading to severe conditions including kidney failure, bone fractures, lung cancer, and other diseases [2,3,4,5].

As primary producers, plants are highly susceptible to Cd stress, which significantly disrupts their growth and development. Cd exposure markedly impairs plant morphology, as evidenced in species such as *Melia azedarach* L., *Toona ciliata* Roem. and *Populus* seedlings, where it significantly reduces key growth parameters such as leaf number and size, root length, seedling height, basal diameter, and biomass, accompanied by leaf chlorosis [6,7,8]. Furthermore, Cd adversely affects photosynthetic processes; for instance, in *Populus* seedlings, it leads to declines in chlorophyll a, chlorophyll b, and total chlorophyll content, as well as reductions in the net photosynthetic rate (Pn), stomatal conductance (Gs), and transpiration rate (Tr) [9,10]. Cd stress also triggers the overproduction of reactive oxygen species (ROS), such as superoxide anions (·O_2_^−^) and hydrogen peroxide (H_2_O_2_) [11], which have been documented in *Arabidopsis thaliana*, *Brassica napus*, and *Malus* spp. [12,13,14]. This oxidative burst promotes lipid peroxidation, resulting in the accumulation of malondialdehyde (MDA), as observed in species like *Cinnamomum camphora* [15] and *Davidia involucrata* [16]. In response, plants typically activate antioxidant defense systems, upregulating enzymes and metabolites such as glutathione, ascorbic acid, proline, and polyphenols to scavenge ROS [17].

Auxin, or indole-3-acetic acid (IAA), is a pivotal plant hormone that regulates nearly all aspects of growth, development, and environmental adaptation by precisely modulating cell growth [18]. Cd stress is known to disrupt endogenous auxin homeostasis. For instance, mild Cd stress induces IAA synthesis and accumulation in barley root tips, whereas severe stress rapidly depletes IAA levels [19]. A similar suppression of root IAA content under Cd stress has been reported in *Sorghum bicolor* [20]. Such fluctuations in IAA concentration directly regulate auxin signaling pathways. Central to this transduction process are Aux/IAA proteins, which function as multigene families and have been characterized in species such as *Arabidopsis thaliana* [21], *Populus tomentosa* [22], *Oryza sativa* [23], *Zea mays* [24], and *Solanum lycopersicum* [25]. These transcriptional regulators exert broad influence on plant morphology; for example, mutations in *PtrIAA14.1* in Populus cause curled leaves, increased branching, and reduced fertility [26]. Similarly, stable expression of Eucalyptus *IAA4* in Arabidopsis disrupts root gravitropism, impaired vascular development (disappearance of xylem fibers), and delays fiber growth [27].

MYB transcription factors represent one of the largest gene families in plants and are well-established regulators of hormone signaling pathways critical for stress adaptation [28,29], fulfilling multiple roles in abiotic stress responses [30]. In horticultural species, MYBs are increasingly implicated in abiotic stress tolerance; for example, Camellia sinensis *CsMYB4a* directly interacts with the auxin signaling repressor CsAUX/IAA4 to regulate developmental processes [31], highlighting a direct mechanistic link to hormone transduction. Furthermore, certain MYBs modulate the expression of metal chelators, antioxidant enzymes, and hormone-responsive genes, thereby orchestrating defense mechanisms against heavy metal stress [28]. For instance, MYB75 in *Arabidopsis* synergistically enhances cadmium tolerance by regulating ROS homeostasis [32], while MYB genes in Coptis chinensis have been directly implicated in heavy metal resistance [33]. Moreover, the auxin response module AtIAA19-AtARF7, which is regulated by AtMYB77, is a key determinant of lateral roots and hypocotyl development in Arabidopsis [34]. The regulatory role of MYB TFs in coordinating these complex responses is increasingly recognized as a central component of plant stress adaptation [28]. This accumulating evidence shows the pivotal role of MYB transcription factors in mediating hormone-driven heavy metal stress responses in horticultural plants.

*Ilex verticillata*, a deciduous shrub of the Aquifoliaceae family, has seen rapidly expanding cultivation in China since its introduction in 2006, with its current planting area exceeding 1200 hectares [35,36,37,38]. While valued horticulturally for its persistent winter fruit, its physiological responses to environmental stressors remain poorly characterized. However, the physiological and molecular mechanisms underlying their response to heavy metal stress, particularly the core signaling network regulating its tolerance, remain uncharacterized. This study aims to fill this critical knowledge gap. We not only systematically evaluated the physiological effects of cadmium stress on the growth, photosynthesis, and oxidative homeostasis of *I. verticillata* but, more importantly, integrated transcriptomic and molecular biology approaches to investigate how cadmium stress reshapes its endogenous auxin signaling pathway. The novelty of this work is twofold: (1) it provides the first dynamic transcriptomic profile of *I. verticillata* under cadmium stress, pinpointing the central role of auxin signaling; and (2) most significantly, it identifies and validates a direct physical interaction between the transcription factor IvMYB77 and six nuclear loci of IvIAA proteins. This reveals an unprecedented ‘IvMYB77-IvIAA’ regulatory module, offering a new molecular perspective on how plants directly couple hormone signaling through transcription factors to mitigate cadmium stress. This discovery deepens the understanding of cadmium tolerance in *I. verticillata* and provides potential theoretical targets for cultivating stress-resistant ornamentals or guiding ecological restoration using key genetic elements.

## 2. Results

### 2.1. Changes in Growth of Ilex verticillata

**Figure 1:**
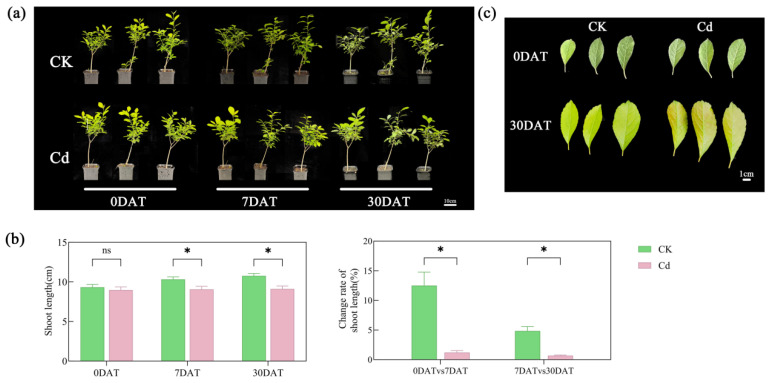
Cadmium stress inhibits growth and morphology of *Ilex verticillata*. (**a**) Representative images showing whole-plant phenotypic changes at 0, 7, and 30 days after treatment. (**b**) Length measurements of the young stems and corresponding changes in growth rates. (**c**) Morphological changes in leaves, with red spots appearing under Cd stress by day 30. Data are shown as mean ± SEM (n = 3). Significant differences between control and Cd-treated groups at each time point were determined by Duncan’s multiple range test (* *p* < 0.05; ns, not significant).

To assess the impact of cadmium (Cd) stress on the growth of *Ilex verticillata*, morphological changes were observed on days 0, 7, and 30 post-treatments (Figure 1a), and the lengths of young stems were measured (Figure 1b). On day 0, there was no significant difference in stem length between the treatment and control groups. However, on day 7 and day 30, the stem length of the Cd-treated plants was significantly reduced by 12.14% and 15.32%, respectively, compared to the control. Furthermore, the stem elongation rate in the control group substantially exceeded that of the treatment group during the 0–7 day and 7–30 days intervals. Leaf morphology was also examined on days 0, 7, and 30 (Figure 1c). While both groups exhibited similar leaf morphology on day 0, red spots appeared on the leaves of the treated group by day 30.

### 2.2. Changes in Photosynthetic Parameters and Chlorophyll Content

**Figure 2:**
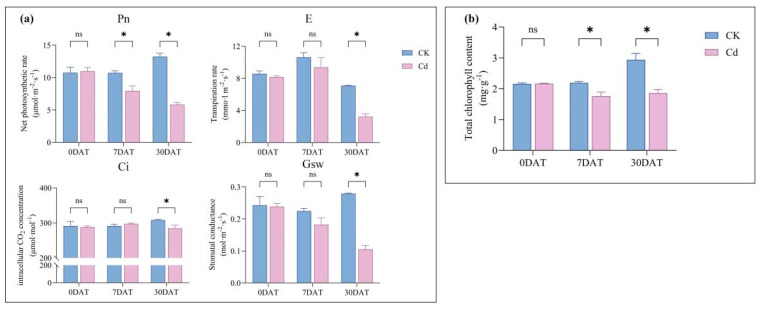
Impact of cadmium stress on photosynthetic parameters in *Ilex verticillata*. (**a**) Gas exchange parameters: net photosynthetic rate (Pn), transpiration rate (E), intercellular CO_2_ concentration (Ci), and stomatal conductance (Gs). (**b**) Total chlorophyll content. Data represent the mean ± SEM (n = 3). Significant differences between the control and Cd-treated groups at each time point were assessed by Duncan’s multiple range test. An asterisk (*) indicates a significant difference at *p* < 0.05; “ns” denotes not significant.

The effects of Cd treatment on photosynthetic performance were evaluated by measuring net photosynthetic rate (Pn), transpiration rate (E), intercellular CO_2_ concentration (Ci), stomatal conductance (Gs), and total chlorophyll content on days 0, 7, and 30 post-treatment (Figure 2). On day 0, no significant differences in Pn were observed between the groups. However, by days 7 and 30, Pn was significantly reduced in the treated group compared to the control group, with decreases of 25.93% and 56.01%, respectively (Figure 2a). For E, no differences were noted on days 0 and 7, but a significant decrease of 54.44% was observed in the treated group on day 30. Ci remained similar on days 0 and 7 but was 7.56% lower in the group treated on day 30. Gs also showed no significant differences on days 0 and 7, but on day 30, it decreased by 62.30% in the treated group (Figure 2a). Total chlorophyll content did not differ between groups on day 0; however, on days 7 and 30, chlorophyll levels in the treated group were 19.61% and 36.73% lower, respectively, compared to the control group (Figure 2b).

### 2.3. Changes in Main Physiological and Biochemical Indices of Roots

**Figure 3:**
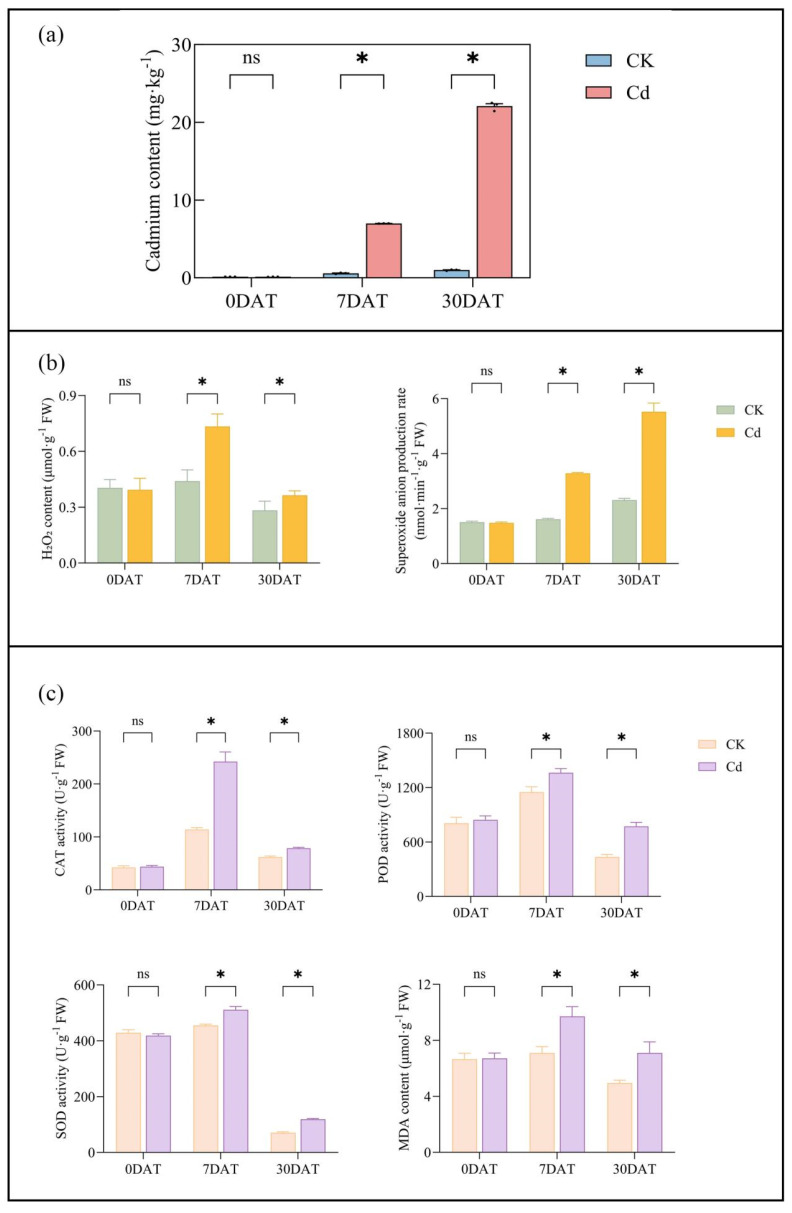
Physiological and biochemical responses of *Ilex verticillata* roots to cadmium stress. (**a**) Cadmium accumulation in root tissues. (**b**) Levels of H_2_O_2_ and the production rate of superoxide anion (·O_2_^−^). (**c**) Activities of key antioxidant enzymes, catalase (CAT), peroxidase (POD), and superoxide dismutase (SOD), and the content of malondialdehyde (MDA). Data are presented as mean ± SEM (n = 3). Significant differences between treatment and control groups at each time point were determined by Duncan’s multiple range test. Asterisks (*) denote a significant difference at *p* < 0.05; “ns” indicates not significant.

The physiological and biochemical responses of *Ilex verticillata* roots to Cd stress were evaluated by measuring catalase (CAT), peroxidase (POD), superoxide dismutase (SOD) activities, MDA content, and cadmium content in root system on days 0, 7, and 30 post-(Figure 3a). On day 0, there were no differences in H_2_O_2_ content among the groups, but on days 7 and 30, it increased by 66.74% and 28.65%, respectively. The superoxide anion production rate showed no differences among groups on day 0 but increased by 103.60% and 139.10% on days 7 and 30, respectively (Figure 3b). On day 0, CAT activity showed no significant differences between groups. However, by days 7 and 30, CAT activity in the treated group increased by 112.58% and 26.91%, respectively, compared to the control group (Figure 3c). Similarly, POD activity showed no differences on day 0 but increased by 18.56% on day 7 and 77.12% on day 30 in the treated group. SOD activity remained unchanged on day 0 but increased by 10.97% and 67.53% on days 7 and 30, respectively. MDA content was significantly higher in the treated group, increasing by 37% and 43.12% on days 7 and 30, respectively (Figure 3c).

### 2.4. RNA-Seq and Mapping to the Reference Genome

**Figure 4:**
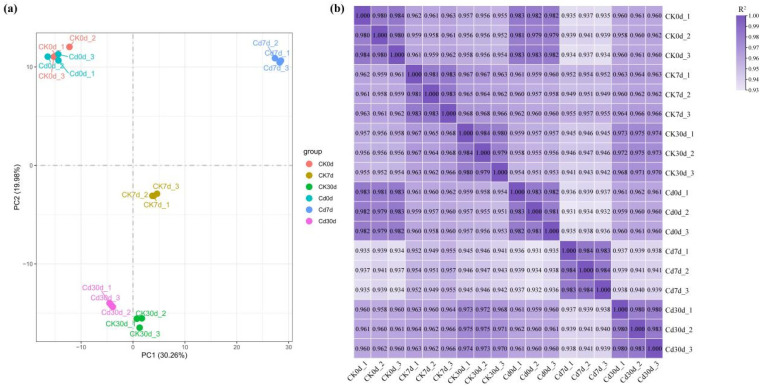
Transcriptomic analysis of *Ilex verticillata* roots under cadmium stress. (**a**) Principal component analysis (PCA) plot showing the separation of samples collected at 0 (Cd0), 7 (Cd7), and 30 (Cd30) days post-treatment. (**b**) Pearson correlation matrix heatmap of gene expression profiles across all samples.

To elucidate the molecular responses of *Ilex verticillata* to Cd stress, transcriptome sequencing was performed on root samples collected at 0, 7, and 30 days post-treatment. A total of 112.46 Gb of clean data was generated from 15 samples, with each sample contributing more than 5.79 Gb of data and a Q30 base quality score above 90%. The clean reads were aligned to the Ilex polycarpa reference genome (Accession: NGDC-GWHBDNW00000000), resulting in an overall alignment rate of 83.92–93.13% (Supplementary Table S1). Uniquely mapped reads accounted for 61.94–64.51%, while multiple mapped reads accounted for 1.83–2.00% (Supplementary Table S1). Principal component analysis (PCA) revealed clear separation of the transcriptomic profiles among the different treatment time points, with the first two principal components (PC1 and PC2) explaining 30.26% and 19.98% of the total variance, respectively (Figure 4a). This indicates that Cd stress significantly altered the global gene expression patterns over time. Furthermore, correlation analysis demonstrated high reproducibility among biological replicates within each group (Figure 4b).

### 2.5. Transcriptomic Profiling of Ilex Verticillata Roots Under Cadmium Stress

**Figure 5:**
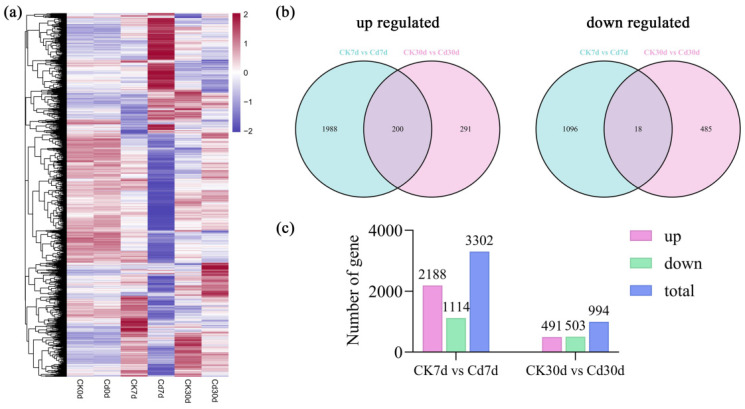
Transcriptional profiling of *Ilex verticillata* roots under cadmium stress: (**a**) Heatmap showing hierarchical clustering of 3750 differentially expressed genes (DEGs) based on Z-score (rows) normalized expression values across treatment time points. (**b**) Venn diagram illustrating the overlap of DEGs between 7-day and 30-day Cd treatment groups. (**c**) Bar plot quantifying the number of upregulated and downregulated DEGs identified at each time point.

A heatmap of Z-score normalized expression values revealed distinct clustering of 3750 differentially expressed genes (DEGs) in *Ilex verticillata* roots under cadmium stress across different treatment periods (Figure 5a). Venn analysis showed that a core set of genes exhibited consistent regulation, with 200 being upregulated and 18 downregulated at both 7 and 30 days post-treatment (Figure 5b). The dynamic nature of the transcriptional response was evident from the number of DEGs at each interval: 3302 DEGs were detected on day 7 (2188 upregulated, 1114 downregulated), whereas 994 DEGs were identified on day 30 (491 upregulated, 503 downregulated) (Figure 5c).

### 2.6. Function Annotation and Enrichment Analysis of DEGs

**Figure 6:**
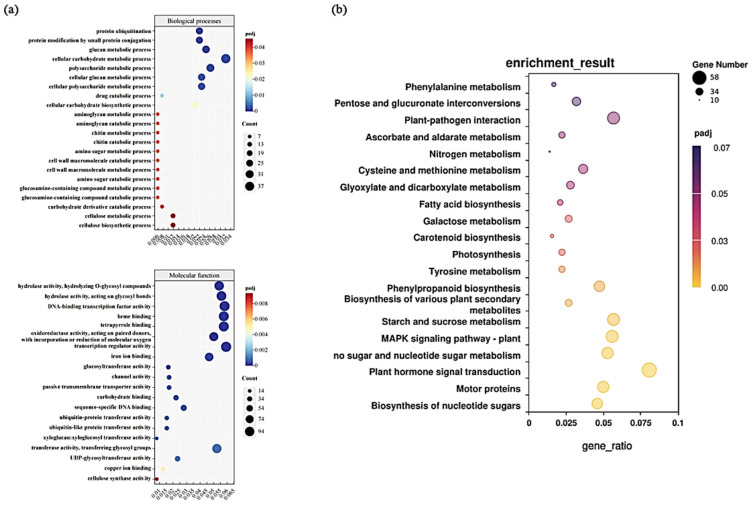
Functional enrichment analysis of differentially expressed genes (DEGs) in *Ilex verticillata* roots under cadmium stress. (**a**) Gene Ontology (GO) enrichment analysis of DEGs (showing the top significantly enriched terms in biological process and molecular function categories). The color gradient from blue to red represents the adjusted *p*-value (padj) from highest to lowest significance. (**b**) Kyoto Encyclopedia of Genes and Genomes (KEGG) pathway enrichment analysis of DEGs. The color gradient from yellow to purple indicates the padj value from highest to lowest significance.

A total of 3750 differentially expressed genes (DEGs) identified under cadmium (Cd) stress at different time points were subjected to functional annotation and enrichment analysis. Gene Ontology (GO) annotation highlighted the top 20 most enriched biological processes and molecular functions (Figure 6a). Within the biological process category, DEGs were significantly enriched in pathways such as “protein ubiquitination,” “small protein conjugation,” “glucan metabolic process,” “cellular carbohydrate metabolic process,” and “polysaccharide metabolic process.” For molecular functions, significant enrichment was observed in “hydrolase activity, hydrolyzing O-glycosyl compounds,” “hydrolase activity acting on glycosyl bonds,” “DNA-binding transcription factor activity,” “heme binding,” and “tetrapyrrole binding”.

Similarly, Kyoto Encyclopedia of Genes and Genomes (KEGG) pathway analysis identified the top 20 enriched pathways (Figure 6b). DEGs were significantly enriched in pathways such as “nucleotide sugar biosynthesis,” “motor proteins,” “plant hormone signal transduction,” “amino sugar and nucleotide sugar metabolism,” “MAPK signaling pathway,” “starch and sucrose metabolism,” “biosynthesis of secondary metabolites,” and “phenylpropanoid biosynthesis”.

### 2.7. Cadmium Stress Alters Auxin Homeostasis and Signaling

**Figure 7:**
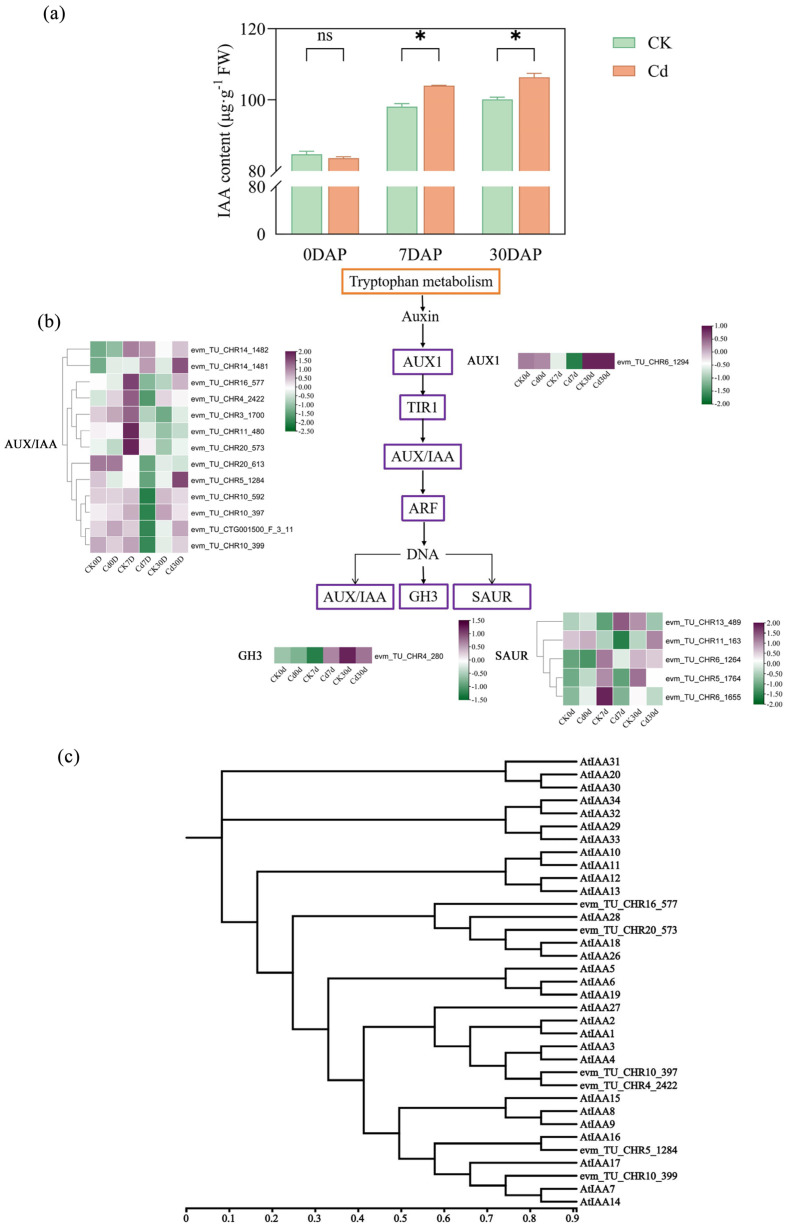
Cadmium stress alters auxin homeostasis and signaling in *Ilex verticillata* roots. (**a**) Endogenous indole-3-acetic acid (IAA) content. (**b**) Heatmap showing expression patterns of DEGs involved in auxin metabolism and signaling pathways. (**c**) Phylogenetic analysis of IvIAA proteins from *Ilex verticillata* and their orthologs in *Arabidopsis thaliana*. Data in (**a**) represent mean ± SEM (n = 3). Significant differences between control and Cd-treated groups at each time point were determined by Duncan’s multiple range test (* *p* < 0.05; ns, not significant).

Endogenous indole-3-acetic acid (IAA) levels were quantified on days 0, 7, and 30 post-treatments based on morphological and transcriptomic analyses. On day 0, no significant differences in IAA levels were observed between the treatment and control groups. However, on days 7 and 30, IAA levels in the treatment group were significantly higher than those in the control group, with improvement of 6.07% and 6.15%, respectively (Figure 7a). KEGG pathway analysis revealed 19 DEGs associated with auxin signaling, primarily involving auxin-responsive factors such as AUX/IAA and SAUR. Most of these genes were significantly upregulated in the treatment group on day 7, with a similar trend observed on day 30 (Figure 7b).

Based on protein annotation, six *IvIAA* genes were identified from the DEGs in the transcriptome data. By constructing an evolutionary tree with the IAA gene family of *Arabidopsis thaliana*, these genes are named *IvIAA4a*, *IvIAA4b*, *IvIAA14*, *IvIAA16*, *IvIAA18*, and *IvIAA28* (Figure 7c).

### 2.8. Molecular Cloning and Sequence Analysis of IvIAA Genes from Ilex verticillata

The six identified *IvIAA* genes were successfully cloned, and their characteristics and physicochemical properties were analyzed (Table 1). The length of CDS is 186–1053 bp, encoding 191–374 amino acids, and the molecular weight is 21,263.16–41,565.68 Da. The isoelectric point (pI) is between 5.27 and 8.88. The instability index ranges from 36.50 to 53.84, among which IvIAA4a and IvIAA14 are stable proteins (index < 40), and the rest of the proteins are unstable proteins (index > 40). The hydrophilicity index of all IvIAA proteins was negative, indicating their hydrophilicity. Subcellular localization confirmed the nuclear localization of all six proteins.

**Figure 8:**
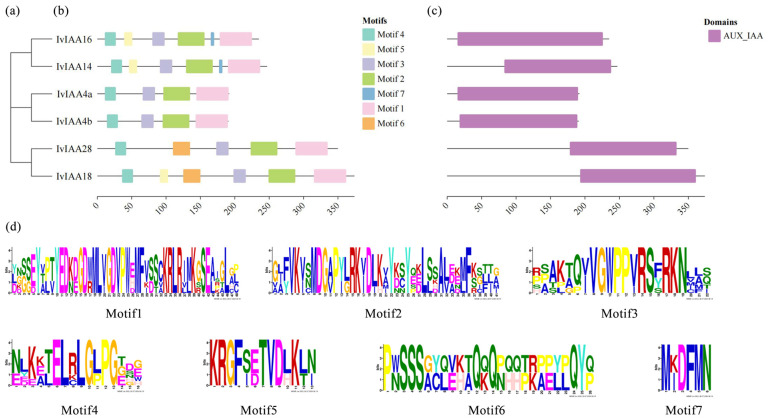
Sequence and structural characterization of IvIAA proteins in *Ilex verticillata*: (**a**) phylogenetic tree showing the evolutionary relationship of six IvIAA proteins. (**b**) Distribution of conserved motifs identified in IvIAA protein sequences. (**c**) Conserved AUX/IAA domains across the identified proteins; (**d**) Sequence logos of the conserved motifs (shown in panel (**b**)).

Conserved motifs and domains in the IvIAA proteins were analyzed using the MEME tool (Figure 8b). Six motifs were identified, with Motif 1, Motif 2, Motif 3, and Motif 4 present in all proteins. Motif 6 was found in IvIAA18 and IvIAA28, while Motif 7 was present in IvIAA14 and IvIAA16. Additionally, Motif 5 was identified in IvIAA14, IvIAA16, and IvIAA18. Domain analysis revealed that all six proteins contained the AUX/IAA domain (Figure 8c).

### 2.9. Subcellular Localization of IvIAA Proteins and Their Interaction with IvMYB77

**Figure 9:**
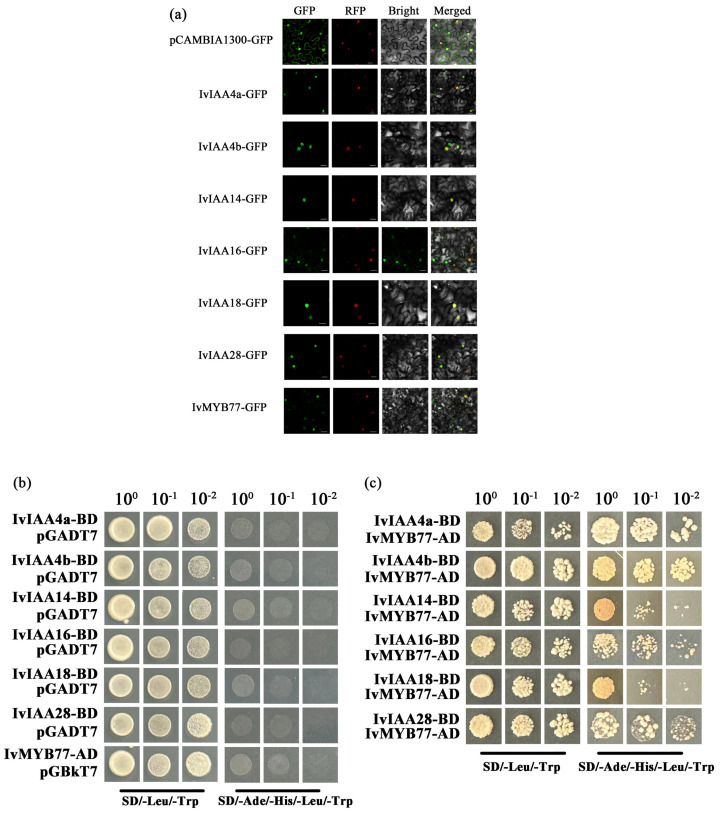
Subcellular localization and protein–protein interactions of IvIAA and IvMYB77. (**a**) Confocal microscopy images showing nuclear localization of IvIAA-GFP and IvMYB77-GFP fusion proteins in tobacco epidermal cells. Scale bar = 20 µm. (**b**) Yeast two-hybrid assays demonstrating physical interactions between IvMYB77 and various IvIAA proteins. Growth on SD/-Leu/-Trp/-His/-Ade medium indicates positive interactions. (**c**) Schematic summary of the interaction patterns between IvMYB77 and IvIAA proteins.

To further elucidate the functions of IvIAA proteins, the genes encoding *IvIAA4a*, *IvIAA4b*, *IvIAA14*, *IvIAA16*, *IvIAA18*, and *IvIAA28*, as well as the differentially expressed *IvMYB77* gene, were inserted into the pCAMBIA1300 vector and transiently expressed in tobacco (Nicotiana benthamiana). Subcellular localization studies revealed that all proteins were localized in the nucleus (Figure 9a).

The regulatory interactions between IvMYB77 and IvIAA proteins were investigated using yeast two-hybrid assays. The results demonstrated interactions between IvMYB77 and IvIAA4a, IvIAA4b, IvIAA14, IvIAA16, IvIAA18, and IvIAA28 (Figure 9b). These findings were further confirmed through protein–protein interaction analysis (Figure 9c).

**Figure 10:**
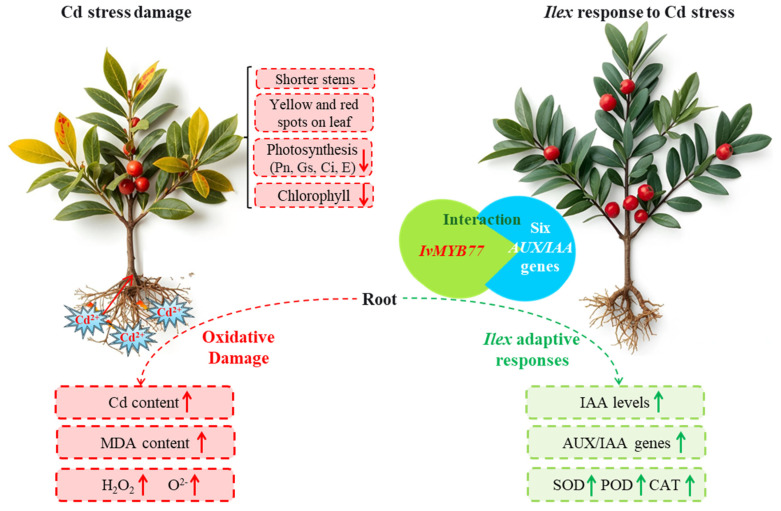
Proposed model illustrating cadmium (Cd) stress response mechanisms in *Ilex verticillata*. Cd exposure induces physiological damage (Red color), including growth inhibition (shorter stems, leaf chlorosis), reduced photosynthetic capacity (Pn, Gs, Ci, E, chlorophyll content), and oxidative stress (elevated Cd, MDA, H_2_O, and O_2_^−^ levels). In response, *I. verticillata* activates a coordinated defense involving increased antioxidant enzyme activities (SOD, POD, CAT), elevated auxin (IAA) levels, and upregulation of AUX/IAA genes. The identified “IvMYB77-IvIAA” regulatory module (comprising six nucleus-localized IvIAA proteins) suggests a potential adaptive mechanism (Green color) for modulating auxin signaling under Cd stress.

Proposed model figure, illustrate that Cd stress inhibited *Ilex verticillata* growth, reducing stem elongation and photosynthetic parameters while inducing oxidative damage (Figure 10). The plant countered Cd stress by enhancing antioxidant enzyme activities (SOD, POD, CAT) and altering auxin signaling, as evidenced by elevated IAA levels and AUX/IAA gene expression. We further identified a potential “IvMYB77-IvIAA” regulatory module, suggesting a mechanism for coordinating auxin-mediated adaptation to Cd toxicity (Figure 10). This integrated response highlights the physiological and molecular strategies employed by *I. verticillata* to mitigate Cd stress.

## 3. Discussion

Cadmium (Cd) pollution remains a significant environmental concern in industrialized regions, presenting a severe threat to agricultural sustainability and ecosystem integrity [39]. *Ilex verticillata*, an ornamental horticultural species introduced to China, has been proposed for nationwide cultivation due to its ecological and aesthetic value [37]. However, a significant knowledge gap exists regarding its physiological and molecular adaptive mechanisms to heavy metal stress, particularly Cd. Addressing this, our study integrated multi-faceted physiological, biochemical, and transcriptomic analyses to decipher the response of *Ilex verticillata* to Cd exposure. Our results demonstrate that Cd stress significantly inhibits stem growth and impairs photosynthetic efficiency, consistent with established phytotoxic effects of Cd in other species [6,7,9]. Furthermore, transcriptomic profiling and auxin quantification demonstrate that the modulation of endogenous auxin signaling is a key component of the Cd stress response in *Ilex verticillata*. Critically, we identified and validated a novel regulatory interaction between the transcription factor IvMYB77 and members of the IvIAA gene family under Cd stress.

The phytotoxic impact of cadmium on photosynthetic machinery is a well-documented phenomenon across diverse plant species, including *Lactuca sativa* [40], *Solanum nigrum* [41], and *Oenanthe javanica* [42]. Our physiological assessment of *Ilex verticillata* not only corroborates these established findings but also delineates a coherent sequence of Cd-induced damage. We observed a significant suppression of key gas exchange parameters including transpiration rate, stomatal conductance, and net photosynthetic rate (Figure 2a), alongside a marked decline in chlorophyll content-when compared to control groups (Figure 2b). These impaired parameters strongly suggests that Cd disrupts both the light-harvesting complexes and the stomatal control of carbon assimilation. Crucially, this photosynthetic impairment is intrinsically linked to a state of oxidative burst, a connection further supported by studies in *Nicotiana tabacum* [43], *Triticum aestivum* [44], and *Betula luminifera* [45]. Our data provides direct evidence for cadmium-induced oxidative stress, showing a significant accumulation of ROS (H_2_O_2_ and ·O_2_^−^) in the root system (Figure 3b). This ROS burst aligns with established models in Arabidopsis and other species where cadmium disrupts redox homeostasis, often visualized via histochemical stains like DAB for H_2_O_2_ [12]. While our assay quantified total root ROS, studies localizing ROS to specific domains (e.g., root elongation zones, vasculature) highlight its dual role as both a cytotoxic agent and a spatial signal influencing growth and defense responses. In our study, the consequent increase in malondialdehyde (MDA) levels (Figure 3c) confirms that this ROS accumulation led to tangible oxidative damage, specifically lipid peroxidation and membrane integrity loss [11]. In response, *I. verticillata* mounts a defense by modulating the activities of core antioxidant enzymes such as superoxide dismutase (SOD), peroxidase (POD), and catalase (CAT) (Figure 3c). However, the persistent oxidative damage and growth inhibition observed indicates that this enzymatic countermeasure is ultimately insufficient to fully neutralize the Cd-provoked oxidative stress [11,16,17]. A key finding of this study was the differential photosynthetic response over time. At 7 days of cadmium stress, the net photosynthetic rate (Pn) of *I. verticillata* decreased significantly (−25.93%), while stomatal conductance (Gs), transpiration rate (E), and intercellular CO_2_ concentration (Ci) remained unchanged (Figure 2a). This specific parameter combination—reduced Pn with stable or elevated Ci—is a classic indicator that the initial inhibition is dominated by non-stomatal factors [46]. We hypothesize that Cd^2+^ directly damages chloroplast ultrastructure, inhibits the activity of photosystem II, or reduces Rubisco efficiency, thereby compromising the biochemical capacity for CO_2_ fixation within the mesophyll [46]. At this early stage, stomatal aperture is not yet the primary constraint on carbon assimilation.

By 30 days, a significant shift occurred: Pn was further suppressed (−56.01%), and this was accompanied by significant declines in Gs, E, and Ci (Figure 2a). This indicates that stomatal limitation has become a major contributing factor. We propose that prolonged Cd toxicity and the associated intensification of oxidative stress (Figure 3b) triggered a systemic plant stress response. Stomatal closure, potentially mediated by ROS and ABA signaling pathways, may serve as an adaptive strategy to reduce water loss and further Cd influx [46]. Therefore, the severe photosynthetic inhibition at 30 days results from the combined action of both non-stomatal and stomatal limitations, reflecting a temporal progression in Cd stress impact from direct biochemical damage to integrated physiological regulation. Collectively, these data provide a mechanistic timeline: Cd exposure first disrupts photosynthetic gas exchange and chlorophyll synthesis, which is closely followed by a significant oxidative burst. This cascade of damage, from initial photosynthetic impairment to sustained oxidative stress, provides a clear physiological explanation for the observed growth inhibition in *Ilex verticillata*.

A notable finding of this study is the impact of Cd on auxin levels in the roots of *Ilex verticillata*. Previous studies in *Arabidopsis thaliana* [47] and *Oryza sativa* [48,49] have established that Cd can dysregulate endogenous IAA levels, the direction and timing of this response are often species- and context-dependent. Our results reveal a distinct species-specific response, demonstrating that auxin levels in roots significantly increased on days 7 and 30 post-treatment compared to controls (Figure 7a). We propose this is an adaptive strategy to re-prioritize root growth and explore uncontaminated soil zones [19,24], a response that differs from the IAA suppression reported in species like Sorghum bicolor [20]. Transcriptomic analysis identified 3750 differentially expressed genes (DEGs; FDR < 0.05, Fold Change ≥ 1.5), many of which were enriched in plant hormone signal transduction pathways, particularly auxin signaling (Figure 6). The pronounced enrichment within the auxin signaling pathway indicates that the observed IAA fluctuation is not a passive consequence of toxicity but an active, regulated process [19]. Therefore, the coordinated upregulation of auxin-responsive genes provides strong evidence for an active re-programming of the auxin signaling network to mediate the complex stress response of *I. verticillata* to cadmium toxicity.

Our investigation identified six nucleus-localized AUX/IAA genes in *Ilex verticillata* that were transcriptionally reprogrammed under Cd stress. This finding aligns with the established role of the AUX/IAA family as a key regulatory node, integrating diverse abiotic stress signals, as extensively documented in Arabidopsis [50]. Previous research on Arabidopsis has demonstrated that Cd stress induces substantial expression variations in AUX/IAA family members, observed in both wild-type Arabidopsis and methylation-deficient mutants [51]. The differential expression of the six IvIAA genes likely represents a direct transcriptional response to the Cd-induced perturbation of auxin homeostasis we observed. Consequently, our data establishes these IvIAA genes as central mediators, translating the altered auxin landscape into specific downstream adaptive or compensatory responses within the auxin signaling network.

Beyond the auxin-responsive genes themselves, our study provides crucial insight into their upstream regulation. We identified the transcription factor IvMYB77, a member of the extensive plant MYB family renowned for its role in abiotic stress responses [52,53], as a key interactor with the core auxin signaling machinery. While MYB factors like AtMYB77 in Arabidopsis are known to interact with ARF transcription factors to influence development [28], and ARFs themselves dimerize with AUX/IAA repressors [54], a direct MYB-AUX/IAA interface has remained largely unexplored. In our study, we discovered that the differentially expressed IvMYB77 physically interacts with multiple nucleus-localized IvIAA proteins (IvIAA4a, IvIAA4b, IvIAA14, IvIAA16, IvIAA18, and IvIAA28) (Figure 9). This discovery unveils a previously underappreciated regulatory layer in the auxin signaling network. We propose a model in which IvMYB77 directly modulates the AUX/IAA-ARF signaling module (Figure 10), thereby fine-tuning the transcriptional output of auxin to orchestrate Cd stress adaptation in *I. verticillata*. While this novel interaction warrants further functional validation in planta, our Y2H data provides a robust foundation for this proposed mechanism and highlight a promising target for future research on heavy metal tolerance.

## 4. Materials and Methods

### 4.1. Plant Materials

The plant materials used in this study consisted of one-year-old *Ilex verticillata* ‘Red Sprite’ cuttings, approximately 40 cm in height, sourced from the Winterberry Base of Runtu Horticulture Technology Company, Hangzhou, China. Prior to treatment, the plants were transplanted into pots and acclimated under greenhouse conditions at Zhejiang A&F University for one month (25 ± 1 °C, 12 h light/dark cycle, 60–70% relative humidity, with watering every two days). For the treatment group, plants were irrigated with a CdCl_2_ solution to achieve a soil Cd concentration of 500 mg/kg. This concentration was selected based on preliminary experiments and literature reports on related species, as it induced significant phenotypic and physiological stress responses suitable for our study. The control group was irrigated with an equal volume of water under identical conditions. Roots and leaves were sampled on 0, 7, and 30 days after treatment, with three biological replicates per group. Samples were immediately frozen in liquid nitrogen and stored at −80 °C.

### 4.2. Plant Growth Observation

Plants with uniform growth were selected, with three biological replicates per group. Growth conditions were observed on days 0, 7, and 30 post-treatment, and photographs were taken to document morphological changes. Additionally, the lengths of the top three young stems (excluding the main stem) were measured at the same time intervals.

### 4.3. Photosynthetic Parameters and Chlorophyll Content Determination

Photosynthetic parameters, including net photosynthetic rate (Pn), transpiration rate (E), intercellular CO_2_ concentration (Ci), and stomatal conductance (Gs), were measured using the Li-6800XT portable photosynthesis system (Li-Cor, Lincoln, NE, USA) between 7:00 and 10:00 a.m. under saturating light conditions on the third leaf from the apex. Total chlorophyll content was extracted using 95% ethanol and quantified according to the protocol described by Li et al. (2000) [55].

### 4.4. Main Physiological and Biochemical Indexes of Roots

Root samples (0.3 g fresh weight) were used for the determination of the following parameters. Cadmium (Cd) content was measured using inductively coupled plasma optical emission spectrometry (ICP-OES) [56]. The hydrogen peroxide (H_2_O_2_) content was determined using the titanium sulfate colorimetric method (Sangon Biotech, Shanghai, China, D799774). The production rate of superoxide anion (·O_2_^−^) was assayed by the hydroxylamine oxidation method (Sangon Biotech, D799772). The activities of catalase (CAT) and peroxidase (POD) were determined by ultraviolet spectrophotometry and the guaiacol colorimetric method, respectively [56]. Superoxide dismutase (SOD) activity was assessed by measuring the photochemical reduction in nitroblue tetrazolium (NBT). The malondialdehyde (MDA) content was determined using the thiobarbituric acid (TBA) method [57].

### 4.5. Measurement of IAA Content

The endogenous indole-3-acetic acid (IAA) content in crushed and ground whole-root samples was quantified using a commercial Plant IAA Enzyme-Linked Immunosorbent Assay (ELISA) Kit (Fanke Industrial Co., Ltd., Shanghai, China), following the manufacturer’s protocol. The assay was based on a double-antibody sandwich principle. Frozen root tissues (0.1 g) were homogenized in liquid nitrogen and extracted according to the kit’s guidelines. The extracted supernatants were assayed alongside a standard curve of known IAA concentrations (5–80 μg/L). The optical density was measured at 450 nm using a microplate reader (Thermo Fisher Scientific, Waltham, MA, USA). Three independent biological replicates, each with technical duplicates, were analyzed per treatment group.

### 4.6. RNA-Seq and Data Analysis

Root samples of *Ilex verticillata* were collected at 0, 7, and 30 days post-treatment for transcriptomic profiling. Total RNA was extracted, and cDNA libraries were constructed by Novogene Co., Ltd. (Beijing, China). RNA integrity was verified using an Agilent 2100 Bioanalyzer (Agilent Technologies, Santa Clara, CA, USA), with all samples exhibiting an RNA Integrity Number (RIN) greater than 8.0. Sequencing was carried out on the Illumina platform. Clean reads were aligned to the reference genome of *Ilex polyphylla* using HISAT2 (v2.0.5). Gene expression levels were quantified as transcripts per million (TPM) with StringTie (1.3.3b). Differentially expressed genes (DEGs) were identified using DESeq2 (1.20.0), applying a threshold of adjusted *p*-value (padj) ≤ 0.05 and an absolute fold change ≥ 1.5. Functional annotation of DEGs was performed through Gene Ontology (GO) enrichment and Kyoto Encyclopedia of Genes and Genomes (KEGG) pathway analyses using NovoMagic online platform (magic.novogene.com).

### 4.7. RNA Isolation, cDNA Synthesis, Gene Cloning, and Bioinformatic Analysis

Seven differentially expressed AUX/IAA genes and one MYB transcription factor were identified from the transcriptome data, and primers were designed for cloning. Total RNA was extracted from *Ilex verticillata* tissues using the FastPure^®^ Universal Plant Total RNA Isolation Kit (Vazyme, Nanjing, China, Cat. RC411). First-strand cDNA was synthesized using the HiScript II 1st Strand cDNA Synthesis Kit (+gDNA wiper) (Vazyme, Cat. R212). Target genes were amplified using 2 × Phanta Flash Master Mix (Dye Plus) (Vazyme, Cat. P520). PCR products were purified with the FastPure Gel DNA Extraction Mini Kit (Vazyme, Cat. DC301-01) and ligated into the pEASY^®^-Blunt Zero Cloning Kit (TranGen Biotech, Beijing, China, Cat. CB501-01). Constructs were transformed into Escherichia coli DH5α competent cells (Accurate Biology, Changsha, China, Cat. AG11806), and positive clones were sequenced by Hangzhou Youkang Biotechnology Co., Ltd. (Hangzhou, China). The protein sequences of the *Arabidopsis thaliana* AUX/IAA family were downloaded from the TAIR (http://www.arabidopsis.org/, accessed on 19 July 2025). The phylogenetic tree of the AUX/IAA protein families from *Arabidopsis thaliana* and *Ilex verticillata* was constructed using the neighbor-joining method in MEGA (11.0.0.155). The physicochemical properties of the proteins were predicted using the Expasy website (https://web.expasy.org/protparam/, accessed on 22 July 2025). Subcellular localization was predicted using the Cell-PLoc website (http://www.csbio.sjtu.edu.cn/bioinf/Cell-PLoc/, accessed on 22 July 2025). The conserved motifs of the proteins were analyzed using the MEME website (https://meme-suite.org/meme/, accessed on 22 July 2025). The conserved domains were analyzed using the Conserved Domain Database (CDD) on the NCBI website (https://www.ncbi.nlm.nih.gov/cdd/, accessed on 22 July 2025).

### 4.8. Subcellular Localization Assays

Identified IAA genes and the MYB transcription factor were inserted into the pCAMBIA1300-GFP vector and transformed into Agrobacterium tumefaciens strain GV3101 (Weidi Biotechnology, Shanghai, China, Code No. AC1001). Transient transformations were performed on 45-day-old tobacco (*Nicotiana benthamiana*) plants. Fluorescence signals were observed in tobacco leaves 48 h post-transformation using a laser confocal microscope (Zeiss LSM880, Oberkochen, Baden-Württemberg, Germany) at excitation wavelengths of 488 nm and 594 nm.

### 4.9. Yeast Two-Hybrid Assays

The MYB transcription factor was cloned into the pGADT7 vector and transformed into yeast strain Y187 (Weidi Biotechnology, Shanghai, China, Code No. YC1020). The IAA genes were cloned into the pGBKT7 vector and transformed into yeast strain Y2H (Weidi Biotechnology, Code No. YC1002). Colonies were cultured on SD/-Leu and SD/-Trp solid media (Coolaber, Beijing, China, Code Nos. PM2203 and PM2253). After streaking on SD/-Leu/-Trp, colonies were transferred to SD/-Leu/-Trp/-His/-Ade plates to validate protein–protein interactions. Negative controls included combinations of pGADT7-IvMYB77 with pGBKT7-IvIAA4a, IvIAA4b, IvIAA14, IvIAA16, IvIAA18, and IvIAA28.

### 4.10. Statistical Analysis

Statistical analyses were performed using IBM SPSS (version 27). Data was evaluated using one-way analysis of variance (ANOVA) followed by Duncan’s multiple range test to determine significance at a 0.05 level. Before conducting downstream analyses, transcriptomic datasets were standardized using Z-score normalization. To explore patterns of similarity and variation among samples, we performed hierarchical clustering analysis (HCA) and principal component analysis (PCA), which facilitated the identification of inter-group differences while minimizing intra-group variability. These analyses were carried out using the OmicShare online platform (http://www.omicshare.com/tools, accessed on 4 November 2025). Additionally, Venn diagrams were constructed with the EVenn web-based application (https://www.bic.ac.cn/EVenn/#/, accessed on 17 October 2025).

## 5. Conclusions

This study demonstrates that Cd stress severely impairs the growth and physiological functions of *Ilex verticillata*, as evidenced by reduced stem elongation, diminished photosynthetic efficiency, and increased oxidative damage. The disruption of auxin homeostasis, coupled with the upregulation of IvIAA genes and their proteins interact with IvMYB77, underscores the critical role of auxin signaling in Cd stress responses. Transcriptomic analysis further revealed the involvement of plant hormone pathways, particularly auxin, in mediating these adaptive mechanisms. These findings not only advance our understanding of Cd stress tolerance in *I. verticillata* but also provide potential targets for enhancing heavy metal resistance in ornamental and crop plants. Future research should explore the functional validation of the “IvMYB77-IvIAA” module and its applicability in phytoremediation strategies.

## Figures and Tables

**Table 1:** Physicochemical characterization of identified IvIAA proteins in *Ilex verticillata*.

Gene Name	Locus ID	CDs (bp)	ORF (aa)	PI	MW (Da)	Instability Index	GRAVY	Predicted Location(s)
IvIAA4a	evm_TU_CHR10_397	579	192	5.27	21,643.43	38.91	−0.763	Nucleus
IvIAA4b	evm_TU_CHR4_2422	576	191	5.61	21,263.16	42.3	−0.58	Nucleus
IvIAA14	evm_TU_CHR10_399	744	247	8.55	27,586.96	36.5	−0.536	Nucleus
IvIAA16	evm_TU_CHR5_1284	708	235	7.63	25,986.4	43.62	−0.666	Nucleus
IvIAA18	evm_TU_CHR20_573	1125	374	8.76	41,565.68	53.84	−0.815	Nucleus
IvIAA28	evm_TU_CHR16_577	1053	350	8.88	38,462.53	48.89	−0.642	Nucleus

Note: coding sequence (CDs) length, open reading frame (ORF) size, isoelectric point (pI), molecular weight (MW), grand average hydropathicity (GRAVY).

## Data Availability

The original contributions presented in this study are included in the article/Supplementary Materials. Further inquiries can be directed to the corresponding authors.

## Supplementary Materials

The following supporting information can be downloaded at: https://www.mdpi.com/article/10.3390/plants15020277/s1, Table S1: Sequencing data and alignment results with the reference genome; Table S2: Primers used in this study.
